# Human Stem Cell Responses and Surface Characteristics of 3D Printing Co-Cr Dental Material

**DOI:** 10.3390/ma12203419

**Published:** 2019-10-18

**Authors:** Boldbayar Ganbold, Seong-Joo Heo, Jai-Young Koak, Seong-Kyun Kim, Jaejin Cho

**Affiliations:** 1 Department of Prosthodontics, School of Dentistry, Seoul National University, 101 Daehak-ro, Jongno-gu, Seoul 03080, Korea; 2 Department of Prosthodontics and Dental Research Institute, Seoul National University Dental Hospital, School of Dentistry, Seoul National University, 101 Daehak-ro, Jongno-gu, Seoul 03080, Korea; 0504heo@snu.ac.kr (S.-J.H.); young21c@snu.ac.kr (J.-Y.K.); 3 Department of Dental Regenerative Biotechnology, School of Dentistry, Seoul National University, 101 Daehak-ro, Jongno-gu, Seoul 03080, Korea

**Keywords:** 3D printing, stem cell, cobalt-chrome, dental alloy, milling

## Abstract

Recently, the selective laser melting (SLM) method of manufacturing three dimensional (3D) dental prosthetics by applying a laser to metal powder has been widely used in the field of dentistry. This study investigated human adipose derived stem cell (hADSC) behavior on a 3D printed cobalt-chrome (Co-Cr) alloy and its surface characteristics and compared them those of a nickel-chrome (Ni-Cr) alloy. Alloys were divided into four groups according to the material and manufacturing methods. Co-Cr disks were manufactured with three different methods: a conventional casting method, a metal milling method, and an SLM method. Ni-Cr disks were manufactured with a conventional casting method. The surface roughness and compositions of the disks were assessed. hADSCs were then cultured on the disks. Cell morphologies on the disks were analyzed by a field emission scanning electron microscope (FE-SEM). Cell proliferation was assessed with a bromodeoxyuridine (BrdU) assay kit. Cell viability was evaluated with a water-soluble tetrazolium salt (WST) assay kit. There were no differences in surface roughness between all groups. The cells were well attached to the disks, and morphologies of the cells were similar. The cell proliferation and viability of the Ni-Cr disks were significantly lower than the other groups. However, the Co-Cr disks showed no differences in their different fabricating methods. In conclusion, the biocompatibility of 3D printed Co-Cr alloys showed comparable results compared to that of the conventional casting method, and these alloys were more biocompatible than Ni-Cr alloys.

## 1. Introduction

Cobalt-chrome (Co-Cr) and nickel-chrome (Ni-Cr) have been used as alternatives to gold alloys for frames of metal ceramic crowns. Co-Cr is commonly used in orthopedic implants as well [1]. However, cytotoxicity is reported when Ni-Cr is used as a dental alloy [2]. Moreover, cytotoxicity induced by Ag, Co, Cr, In, and Cu suggests a necessity of design change of the dental alloy [3]. Recently, the selective laser melting (SLM) method for constructing three dimensional (3D) dental prosthetics by applying a laser to metal powder has been broadly studied in the field of dentistry [4]. The SLM technique is an additive manufacturing (AM) method that forms the materials layer by layer, while the subtractive manufacturing (SM) technique involves cutting solid blocks. Milling such hard metal causes a waste of milling tools due to undue stress. Therefore, soft metal blocks have emerged as a solution [5]. However, the precision of the SM method of milling soft metal blocks could be varied by vibration of the milling tool [6]. The SLM method could overcome such problems, applying a direct fiber laser to the metal powder [7]. A previous study reported that SLM technology made implant screws more easily, and the screws made by SLM showed precise insertion during spinal surgery than traditionally produced screws [8]. Recently, Oladapo et al. [9,10] introduced the method of combining two materials using AM and analyzed nanoparticles and various resin behaviors to improve products manufactured with AM technologies. In mechanical perspectives, Co-Cr dental restorations made by SLM can provide properties comparable to or better than those fabricated with conventional casting and computer-aided design/computer-aided manufacturing (CAD/CAM) milling techniques [11].

A year of clinical research focused on the influence of Co-Cr frameworks of implant-supported fixed partial dentures (FPDs) made at the implant level on marginal bone loss compared with those made at the abutment level. The implant-level frameworks had more marginal bone loss than the abutment-level frameworks [12]. When a framework is made at the implant level, it is in direct contact with peri-implant tissue, which could be an important factor for biological complications [13]. However, clear evidence on the soft and hard tissue response to Co-Cr frameworks is still lacking [14]. Therefore, studying the biocompatibility of a framework is crucial for developing the alloys in dentistry.

Stem cells have been used to analyze biocompatibilities of dental implants and alloys [15,16]. The cell response of titanium implants manufactured with the SLM method was also evaluated in another study [16]. In the study that compared the influences of adipose-derived stem cells (ADSCs) and dental pulp stem cells (DPSCs) on bone formation, ADSCs proved to induce more bone formation than DPSCs did in vivo. This result suggests that ADSCs have a positive effect on bone regeneration [17]. ADSCs also show a similar response to other stem cells from different origins-responses that were somatic and that were related to bone marrow and osteogenesis [18]. Therefore, human ADSCs were used in this study.

The purpose of this study was to assess the biocompatibility of 3D printed Co-Cr alloys and to compare it to Co-Cr alloys fabricated with the conventional casting method and milling technique. Ni-Cr alloys produced by the conventional casting method were compared as well.

## 2. Materials and Methods

### 2.1. Cobalt-Chrome and Nickel-Chrome Specimen Preparation and Analyses of Surface Characteristics

A total of 52 disks, 13 each for four groups, were fabricated. As the control group, 13 Ni-Cr (MediS1000, MediFiOn, Daejeon, Korea) disks were fabricated with the conventional casting technique. For the test group, 13 Co-Cr (StarLoyC, Degudent GmbH, Hanau, Germany) disks were fabricated with the conventional casting method as well. Thirteen Co-Cr disks were manufactured by milling a soft metal block (SMB, Soft metal, LHK, Chilgok, Korea) with a dental milling machine (DWX 52D, Roland, Hamamatsu, Japan) for a Co-Cr (milling) group [19]. Thirteen Co-Cr disks were fabricated by a 3D printer (EOS M270, Nobilium, Albany, NY, USA) equipped with a 400-watt fiber laser using Co-Cr powder (EOS Co-Cr SP2, EOS GmbH, Krailling, Germany) for a Co-Cr (3D) group. Each disk had uniform dimensions with a 15-mm diameter and 1-mm thickness. Table 1 shows the element proportions of the alloys.

Before polishing all the disks, surface microstructures were investigated with a field emission scanning electron microscope (FE-SEM) (JSM-7800F, JEOL, Tokyo, Japan) with a voltage of 5 Kv. Disk surfaces were then milled by a lathe machine (TIPL-4, S&T, Changwon, Korea) that rotates the disk about an axis of rotation to polish with a carbide tool bite that is applied to the disk to create a smooth surface. A custom zig was made to fix the disks to the lathe machine. After polishing, all disks were washed with alcohol in an ultrasonic bath and cleaned with distilled water. In the final step, the disks were disinfected in an autoclave. The disks were sputter coated with platinum, and two magnifications of views (×1000, ×5000) were investigated by FE-SEM. Chemical compositions of the disks were assessed by energy dispersive X-ray spectroscopy (EDS) (X-max, Oxford Instruments, Abingdon, UK). Surface roughness was investigated at three points on three specimens for each group with confocal laser scanning microscopy (CLSM) (LSM 800, Zeiss, Obercochen, Germany).

### 2.2. Analyses of hADSC Responses on Co-Cr and Ni-Cr Specimens

#### 2.2.1. hADSC Preparation and Culturing

hADSCs were obtained from 20-year-old female patients at Seoul National University Dental Hospital. The procedure of collecting ADSCs was carried out during the operation for temporomandibular joint treatment. The study was conducted in accordance with the Declaration of Helsinki, and the protocol was approved by the Ethics Committee of School of Dentistry, Seoul National University (IRB No. S-D20190021). hADSCs were cultured in Dulbecco’s Modified Eagle Medium (DMEM, WELGENE, Gyeongsan, Korea) containing 10% fetal bovine serum (FBS, Gibco, Rockville, MD, USA) and 1% Antibiotic-Antimycotic (Thermo Fisher Scientific, Waltham, MA, USA) and were incubated at 37 °C in a humidified atmosphere of 5% CO_2_. Cell culture media were renewed every two days. When the cell population grew to 70~80% confluence, they were washed with Dulbecco’s Phosphate-Buffered Saline (DPBS, WELGENE, Gyeongsan, Korea) and detached from the cell culture by 0.25% trypsin ethylenediaminetetraacetic acid (EDTA, Gibco, Rockville, MD, USA). The cell passaging process was then done until the cells reached Passage 4. Disk specimens were placed at the bottom of a 24-well plate [20]. Cells were directly seeded on the surfaces of Co-Cr and Ni-Cr disks at a density of 1 × 10^4^ cells/disk/well with 1 mL of culture media for each well [21].

#### 2.2.2. hADSC Morphologic Analysis by FE-SEM

After two days of cell culture on the disks, cells were fixed with 2% glutaraldehyde in DPBS for 15 min at room temperature [22]. Cells were stained with 2% osmium tetroxide in PBS for 15 min and dehydrated in an ascending ethanol series. After critical point drying, samples were sputter-coated with 6 nm platinum and then examined in the FE-SEM.

#### 2.2.3. Proliferation Assay by BrdU

Bromodeoxyuridine (BrdU) incorporation assay was done using a BrdU Cell Proliferation Assay kit (Merck KGaA, Darmstadt, Germany) according to the manufacturer’s protocol. After one day of cell culture, the BrdU labeling reagents were added to the well. After 24 h of incorporation of BrdU into deoxyribonuclease (DNA), anti-BrdU and a peroxidase-conjugated secondary antibody were added to the well [23]. Three aliquots of 100 µL mixtures from each cell culture well were transferred to a 96-well plate, and the optical density (OD) values were assessed at 450 nm using a microplate reader (Epotech 2, BioTek, Winooski, VT, USA). Replicate measurements were performed with 3 disks for each group (N = 3).

#### 2.2.4. Cell Viability Assay by WST

After one and four days of cell culturing, cell viability was assessed by the EZ-Cytox cell viability assay kit (DoGenBio, Seoul, Korea) [21]. After washing cells with DPBS at each time point, washing solutions were aspirated. Four hundred microliters of DMEM were then put on each cell culture well, and 40 µL of EZ-Cytox were added to them. A formazan is formed when an EZ-cytox solution reacts to the dehydrogenase of a living cell. The mixtures were incubated for 1 h at 37 °C. Three aliquots of 100 µL mixtures from each cell culture well were then transferred to a 96-well plate [16]. Optical density was measured at 450 nm by a microplate reader. Three disks were used for each group, for each time point (N = 3).

### 2.3. Statistical Analysis

The statistical significance for all data comparisons was assessed by Student *t*-test analysis (SPSS 23, IBM Inc., Armonk, NY, USA). *p*-values less than 0.05 were considered statistically significant.

## 3. Results

### 3.1. Surface Characteristics of the Cobalt-Chrome and Nickel-Chrome Specimens

**Figure 1:**
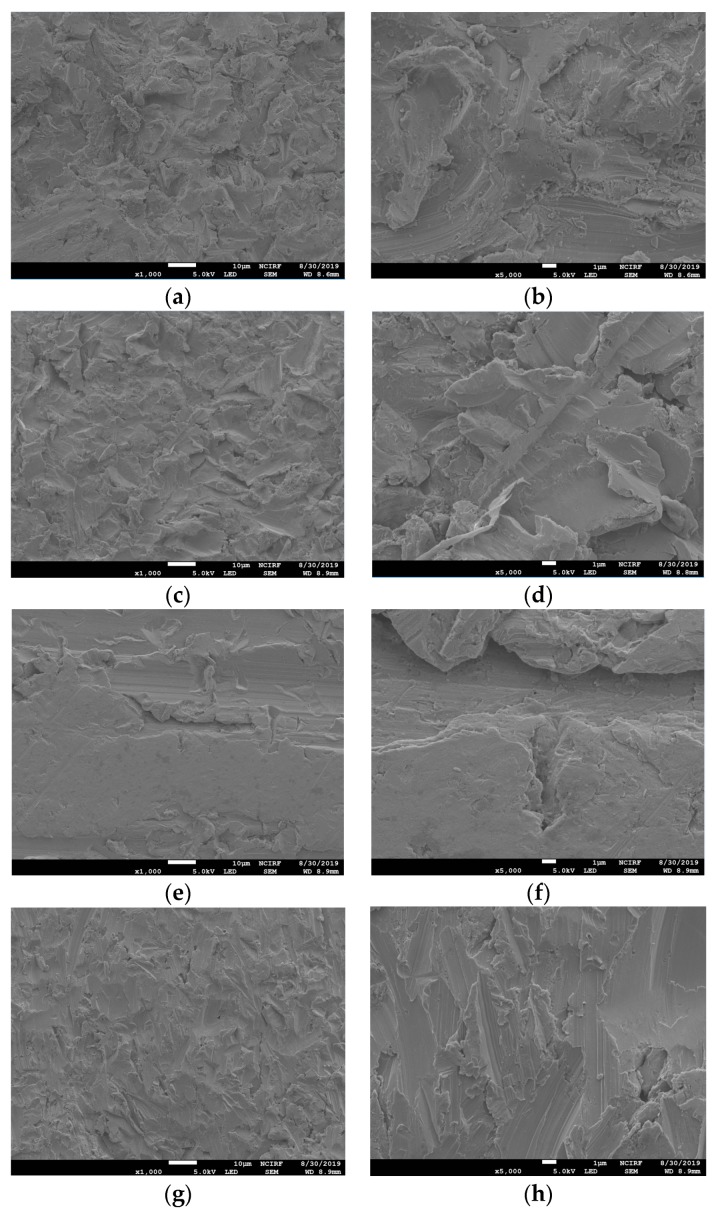
Scanning electron microscope (SEM) images of the surfaces of non-polished Ni-Cr and Co-Cr disks. Ni-Cr group: each row, left to right, original magnification (**a**) 1000×, (**b**) 5000×. Co-Cr (casting) group: (**c**) 1000×, (**d**) 5000×. Co-Cr (milling) group: (**e**) 1000×, (**f**) 5000×. Co-Cr (3D) group: (**g**) 1000×, (**h**) 5000×.

**Figure 2:**
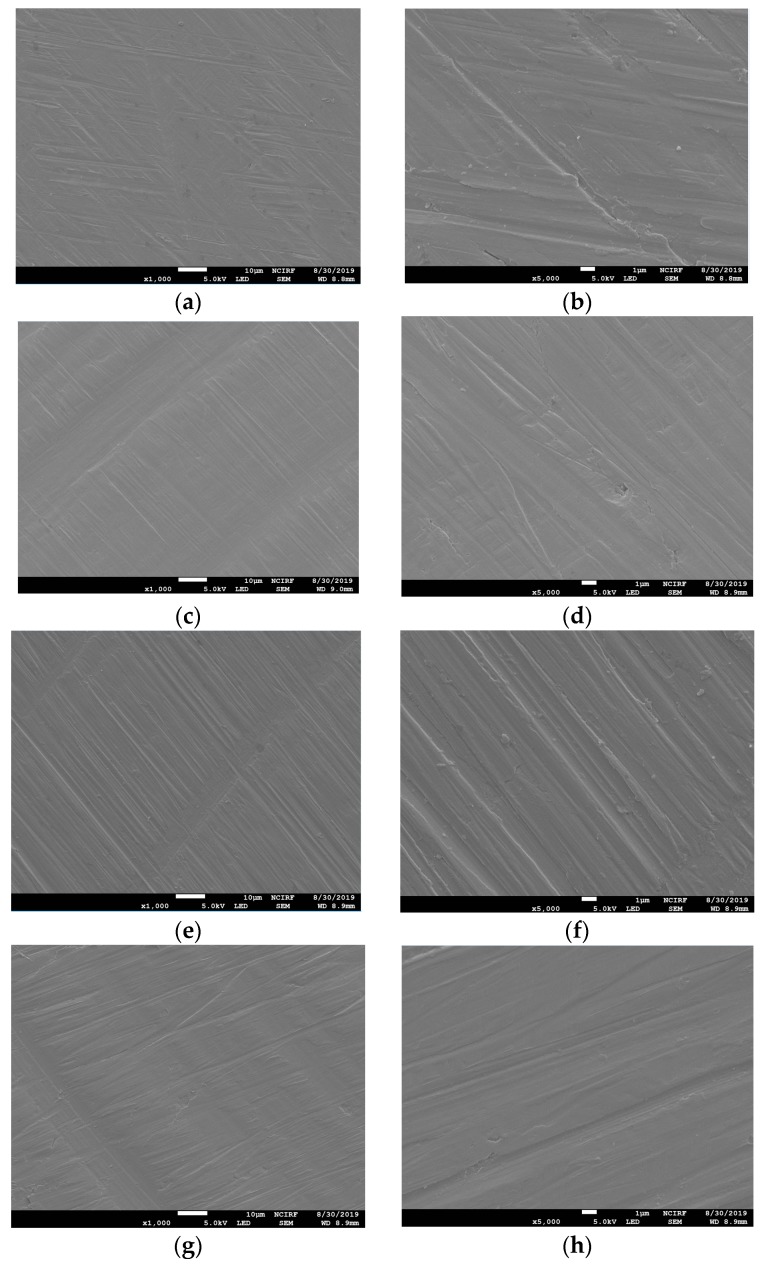
Scanning electron microscope (SEM) images of the surfaces of polished Ni-Cr and Co-Cr disk. Ni-Cr group: each row, left to right, original magnification (**a**) 1000×, (**b**) 5000×. Co-Cr (casting) group: (**c**) 1000×, (**d**) 5000×. Co-Cr (milling) group: (**e**) 1000×, (**f**) 5000×. Co-Cr (3D) group: (**g**) 1000×, (**h**) 5000×.

Figure 1 shows the SEM images of the non-polished disk surface. All disks had irregular rough patterns. Figure 2 shows SEM views of surface images of the disk of the four groups. Surface microstructures had flat surfaces with striped patterns.

**Figure 3:**
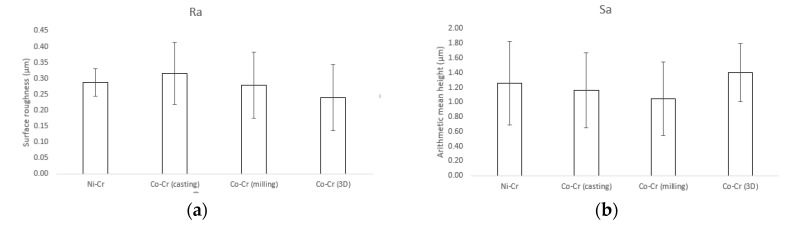
Surface roughness-related parameters obtained by confocal laser scanning microscopy (CLSM). (**a**) Ra (surface roughness), (**b**) Sa (arithmetic mean height deviation from a mean plane).

Results for the surface roughness-related parameters for the polished disks are summarized in Figure 3. The Ra (surface roughness) and Sa (arithmetic mean height deviation from a mean plane) values for Ni-Cr disks were 0.29 ± 0.04 µm and 1.26 ± 0.57 µm, respectively. The Ra and Sa values for Co-Cr (casting) disks were 0.31 ± 0.1 µm and 1.26 ± 0.57 µm, respectively. The Ra and Sa values for Co-Cr (milling) disks were 0.28 ± 0.1 µm and 1.04 ± 0.5 µm, respectively. The Ra and Sa values for Co-Cr (3D) disks were 0.24 ± 0.1 µm and 1.4 ± 0.39 µm, respectively. There was no statistically significant difference between the overall roughness of the four groups.

**Figure 4:**
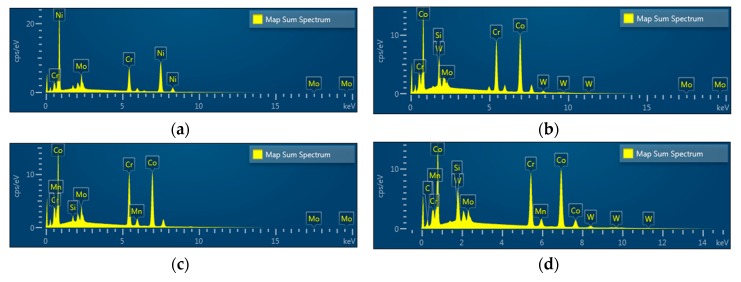
Energy dispersive X-ray spectroscopy (EDS) patterns of the Ni-Cr disk compared with the Co-Cr disk. (**a**) Ni-Cr group, (**b**) Co-Cr (casting) group, (**c**) Co-Cr (milling) group, (**d**) Co-Cr (3D) group.

EDS spectra revealed Ni, Cr, and Mo peaks for Ni-Cr disks and Co, Cr, W, Mo, and Si peaks for Co-Cr (casting) disks. Peaks of Co, Cr, Mo, Si, and Mn were shown in Co-Cr (milling) disks and peaks of Co, Cr, W, Mo, and Si were shown in Co-Cr (3D) disks, confirming that all disks were pure and free from any contamination for further cell experiments (Figure 4).

### 3.2. FE-SEM Assessment of Cell Morphology

**Figure 5:**
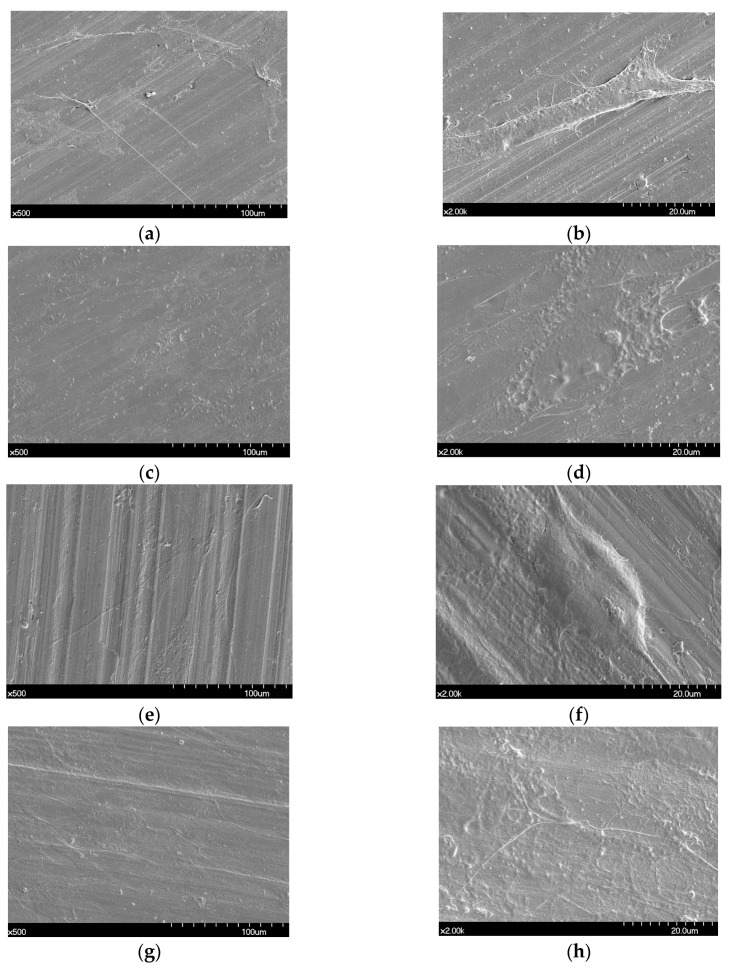
Scanning electron microscopy images showing human adipose derived stem cells (hADSCs) that were seeded on the Ni-Cr disk and the Co-Cr disk. (**a**) Cells seeded on a Ni-Cr disk, original magnification 500×, (**b**) original magnification 2000×, (**c**) cells seeded on a Co-Cr (casting), original magnification 500×, (**d**) original magnification 2000×, (**e**) cells seeded on a Co-Cr (milling) disk, original magnification 500×, (**f**) original magnification 2000×, (**g**) cells seeded on a Co-Cr (3D) disk, original magnification 500×, (**h**) original magnification 2000×.

Proliferated hADSC morphologies are shown in Figure 5. Cells were spread regularly on the surfaces of all disks. A high magnification FE-SEM image (×2000) confirmed that the cells were well attached to all the disks (Figure 5b,d,f,h).

### 3.3. BrdU Assay

**Figure 6:**
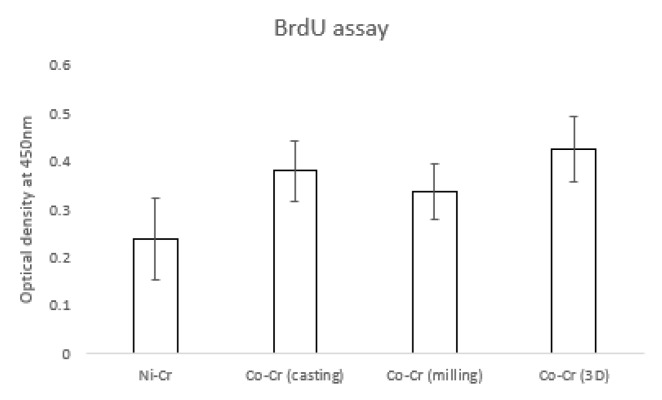
The value of proliferated hADSCs that were incorporated with BrdU. There were significant differences between the Ni-Cr group and the other groups (the bar represents mean ± SD).

Figure 6 shows the hADSC proliferation for the disk. The OD value of the Ni-Cr group (0.23 ± 0.08) was significantly lower than that of other groups. OD values of the Co-Cr (casting) and Co-Cr (milling) groups were 0.38 ± 0.06 and 0.33 ± 0.05, respectively. The OD value was 0.42 ± 0.06 for the Co-Cr (3D) group. There was no significant difference between the Co-Cr (casting) and Co-Cr (3D) groups. However, the Co-Cr (3D) group was significantly higher than the Co-Cr (milling) group.

### 3.4. WST Assay

**Figure 7:**
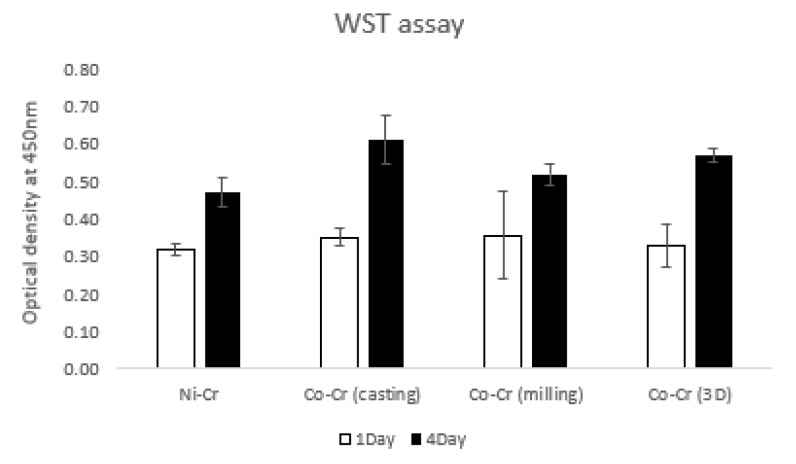
The water-soluble tetrazolium salt (WST) assay of hADSCs cultured on the disks on day 1 and day 4. The value of Ni-Cr was significantly lower than that of other groups both for day 1 and day 4. There were no significant differences between the groups of Co-Cr disks for day 1. There was no significant difference between the Co-Cr (casting) and Co-Cr (3D) groups for day 4 (the bar represents mean ± SD).

Figure 7 shows the values of hADSC viabilities for the disks by the water-soluble tetrazolium salt (WST) assay at day 1 and day 4. On day 1, OD values were 0.32 ± 0.01, 0.35 ± 0.02, 0.36 ± 0.11, and 0.33 ± 0.05 for Ni-Cr, Co-Cr (casting), Co-Cr (milling), and Co-Cr (3D), respectively. The Ni-Cr group was significantly lower than the Co-Cr (casting) group. There were no significant differences among the other groups. On day 4, OD values were 0.47 ± 0.03, 0.61 ± 0.06, 0.52 ± 0.02, and 0.57 ± 0.01 for Ni-Cr, Co-Cr (casting), Co-Cr (milling), and Co-Cr (3D), respectively. The Ni-Cr group was significantly lower than the other groups. The Co-Cr (milling) group was significantly lower than the Co-Cr (casting) and Co-Cr (3D) groups. There was no significant difference between the Co-Cr (casting) and Co-Cr (3D) groups.

## 4. Discussion

This study may be the first attempt to present a comparison of different techniques of manufacturing Co-Cr alloys in relation to cell response while simultaneously comparing it with Ni-Cr alloys. Co-Cr and Ni-Cr dental alloys have been broadly used as prostheses in dental practice. However, there has always been concern about the biocompatibility of metal alloys due to their ion release. It has been confirmed that surface design affects MSC behaviors [24]. In addition, cell response could be affected by the variation of SLM parameters including energy density and the exposure time of lasers on 3D printers [16]. Due to this, we have compared the influences of different techniques of manufacturing Co-Cr alloys on the cell response. In the engineering aspect, vehicle suspension system (VSS) and machine shaft manufactured with 3D technology were analyzed, which could provide guidelines about fatigue strength and design of a machine shaft and VSS [25,26]. It has been reported that Ni-Cr alloys composed of more than 20 wt% chromium show favorable corrosion resistance [27]. Hence, we used the Ni-Cr alloys containing 22 wt% chromium for this study. According to the systematic review study, the Sa values of most implants in the market were between 1.1 and 2.0 µm [28]. Taking this into consideration, we adjusted all the disk surfaces by polishing, resulting in median Sa values between 1.04 and 1.26 µm. There have been trials to improve the bioactivity of metal alloys by surface modification [29,30]. An in vitro study reported that a graphene coating on a Co-Cr alloy enhanced its bioactivity [30]. Also in medical field, the study of surface modification by applying magnetic nanoparticles demonstrated that it could enhance sensitivity and stability of biosensors [31]. Since there were no significant differences in surface roughness between all the disks and no additional surface treatment was done to the disk, the present study was able to demonstrate biocompatibility of the alloys themselves fabricated with different methods with no other variables. In order to show the polishing effect, we have captured SEM views of both surfaces before and after polishing disks. Surface topographies of the disks after polishing result in smooth surfaces with regular striped patterns.

ADSCs are helpful for bone regeneration [32], and a great number of ADSCs can be collected from adipose tissue with properties of steady proliferation and potential differentiation in vitro [33]. ADSCs have also been verified to be a popular stem cell with proved beneficial applications in numerous areas of regenerative medicine [34]. Zhang et al. [18] conducted a study comparing human mesenchymal stem cells (MSCs) derived from multiple sources: dental pulp, periodontal ligaments, gingivae, dental follicles, bone marrow, adipose tissue, and umbilical cord. Bone-marrow and adipose-derived stem cells determined a robust affinity for osteogenesis among stem cells from different origins [18]. We selected ADSCs for this study because of the reasons mentioned above. Cell morphologies on the disks were captured by SEM after two days of cell culture. Cells of all groups had similar morphologies showing a spindle shape with a few pseudopods stretching from its body.

BrdU was incorporated into newly synthesized DNA strands of actively proliferating cells. Following partial denaturation of double stranded DNA, BrdU was detected immunochemically allowing the assessment of the population of cells, which are synthesizing DNA. BrdU has been used since the 1980s to indicate proliferating cells derived from humans and animals [35]. Cell proliferation for Ni-Cr was significantly lower than the other three groups. These results agree with the research that showed the negative effect of Ni-Cr alloys on cell activity [36]. The Co-Cr (casting) group and the Co-Cr (3D) group showed the highest cell proliferation rate, which showed OD values of 0.38 ± 0.06 and 0.42 ± 0.06, respectively. However, the significant difference was only seen between the Co-Cr (milling) (0.33 ± 0.05) and Co-Cr (3D) (0.42 ± 0.06) groups among the three Co-Cr groups.

Stem cell viabilities that we assessed with WST also expressed cytotoxicity of the alloys. A formazan—an orange product formed when high-sensitive WST reacts with dehydrogenase enzyme that exists in active cells—indicates a number of living cells. Water-soluble formazan that does not necessitate an additional solvation step makes the assay less complicated and more sensitive [37]. On day 1, the only group that displayed lower cell viability than the Co-Cr (casting) group was the Ni-Cr group. Cell viability for Ni-Cr was significantly lower than the other three groups for day 4. Nickel-based alloys of dental restoration affected the immune system, which is explained by metal ions being released in the oral cavity in contact with epithelial cells, causing a cytotoxic response [38]. This could be explained by the results of another study reporting that Ni-Cr microparticles had lower biocompatibility than when it was used as a compact alloy [39]. On the other hand, another study [40] revealed similar cytotoxic effects for both Ni-Cr and Co-Cr alloys. However, when Ni-Cr contains Cu, it becomes more toxic than Co-Cr [40].

The highest cell viabilities were found with the Co-Cr (casting) and Co-Cr (3D) group, which showed a significantly higher value than the Co-Cr (milling) group. A previous study showed that the alloy containing 2 wt % tungsten (W) had higher corrosion resistance than those containing no W [41]. The Co-Cr (casting) and Co-Cr (3D) groups containing W might be more corrosion-resistant than the Co-Cr (milling) group with no W component. The contribution to cell viability of the alloys is probably their content of W. In this study, we used Co-Cr soft metal blocks not containing W for the Co-Cr (milling) group because tungsten-free Co-Cr has been widely used in various industries currently. In order to clarify the outcomes, and to explain the effect of its way of fabrication on human cells, all the groups that containing W need to be studied and compared for future studies. These materials are employed to fabricate the framework of the dental prosthesis so they will be covered by porcelain or other veneer material. It has been assured that the Co-Cr (3D) disk showed comparable cell viability to the Co-Cr (casting) disk, and demonstrated higher biocompatibility than the Ni-Cr disks through the BrdU and WST assays. Cautious consideration is needed when using the Ni-Cr alloy for dental prosthodontics. SLM method has a prospect in dental field for fabrication of various dental prostheses since it showed appropriate biocompatibility in the study.

## 5. Conclusions

This study examined the surface characteristics of 3D printed Co-Cr alloy and stem cell responses on the alloy surface. All the groups that contain Co-Cr disks demonstrated better biocompatibility than the Ni-Cr group. The results indicated that the 3D printed Co-Cr alloy has a favorable biocompatibility and can be widely used in dental prosthodontics. Long-term studies about the biocompatibility of metal alloys are needed in the future.

## Figures and Tables

**Table 1:** Compositions of the Co-Cr and Ni-Cr disks.

Groups	Element (wt %)						
	Ni	Co	Cr	Mo	Si	W	Nb
Ni-Cr (casting)	67.4		22	9			
Co-Cr (casting)		59.4	24.5	1	1	10	2
Co-Cr (milling)		56~61	27~30	5~7	1		
Co-Cr (3D)		63.8	24.7	5.1	0.1	5.4	
